# Quantification of Gallic Acidin Fruits of Three Medicinal Plants

**Published:** 2011

**Authors:** Mahdi Vazirian, Mahnaz Khanavi, Yaghoub Amanzadeh, Homa Hajimehdipoor

**Affiliations:** a *Department of Pharmacognosy, Faculty of Pharmacy, Tehran University of Medical Sciences, Tehran,*; b * Traditional Medicine and Materia Medica Research Center and Department of Traditional Pharmacy, Faculty of Traditional Medicine, Shahid Beheshti University of Medical Sciences, Tehran, Iran.*

**Keywords:** Triphala, *Terminalia chebula*, *Terminalia bellerica*, *Phyllanthus emblica*, Gallic acid, Rhodanine assay

## Abstract

Triphala is a traditional herbal formulation consisting of dried fruits originating from three medicinal plants, namely *Terminalia chebula*, *Terminalia bellerica* and *Phyllanthus emblica*. It is used in folk medicine for the treatment of headaches, dyspepsia and leucorrhoea. There are some reports regarding Triphala’s pharmacological effects including its anti-cancer, radioprotective, hypocholesterolaemic, hepatoprotective and anti-oxidant activities. The most important components of these plants are the tannins and gallic acid which they contain. Gallic acid being a compound with tannin structure existing in the Triphala fruit. In this research, the gallic acid content contained in the three plants constituting Triphala was determined. Plant fruits were purchased from available Iranian markets. Milled and powdered fruits from each plant were extracted with 70% acetone and subjected to a reaction with rhodanine reagent in the process forming a colored complex. The complex’s absorbance was measured at 520 nm and the amount of gallic acid was determined using its calibration curve. According to the results, the highest amount of gallic acid was observed in *Phyllanthus embelica* (1.79-2.18%) and the lowest amount was found in *Terminalia chebula* (0.28-0.80%). Moreover, differences between plant samples from different markets places were found to be statistically significant (p < 0.05). These differences can possibly be due to the source of plant preparation, storage condition and period of Triphala storage. In general, the rhodanine assay is a simple, rapid and reproducible method for the standardization of Triphala as gallic acid.

## Introduction

Triphala used as an intestinal tonic is one of the most popular Ayurvedic and Iranian medicines. This product contains dried and powdered fruits from three medicinal plants: *Terminalia chebula *(Halileh), *Terminalia bellerica* (Balileh) and *Phyllanthus emblica *(Amlah) (1). It is used in folk medicine for treatment of headaches, dyspepsia, ascites and leucorrhoea. There are also some reports with regards to Triphala’s pharmacological effects including anti-cancer, radioprotective, hypocholesterolaemic, hepatoprotective and anti-oxidant properties (2-6). Triphala’s fruits are grown mainly in India and Pakistan. They are then exported to other countries such as Iran. 

Plants contain many components with their type and amount differing according to age, geographical location and harvesting process. These factors affect the plants therapeutical properties. This complex situation is further complicated by the combination of herbal ingredients that are used in traditional practice. Therefore, determination of a suitable method for the quality control of herbal formulations is necessary. At the present time it is very difficult to identify the presence of all the ingredients a formulation claims to contain. Therefore in order to control the quality of herbal products, a compound is considered and then quantified as marker (7). Different methodologies have been proposed for standardizing herbal formulations such as HPLC (8-10), GC (11, 12), HPTLC (13), UV/VIS (14), atomic absorption spectroscopy (15) and NMR (16). While for many common quality control assays, spectrophotometry is the method of choice due to its simplicity, low cost and ease of application. Triphala’s fruits contain different components mainly with tannin-like structures. Gallic acid is a phenolic compound that exists in fruits making up Triphala and can be considered as a marker for controlling the quality of them (9, 17). In this research, in order to evaluate the quality of the Triphala available in the Iranian market, the gallic acid content of Triphala’s fruits was determined by means of UV spectroscopy.

## Experimental

*Plant material*

Dried fruit samples of* Terminalia chebula *(including black and yellow ones), *Terminalia bellerica* and *Phyllanhus emblica *were purchased from Iranian herbal markets. Three samples of each plant were prepared from different markets.

*Chemicals*

Gallic acid reference standard and rhodanine were purchased from Sigma (Germany). Other chemicals and solvents were obtained from Merck (Germany).

*Plant extraction*

100 g of fruits from each plant were collected randomly and finely grounded. 10 mL of aqueous acetone (70%) was added to 100-400 mg of the powder and then suspended in an ultrasonic water bath for 20 min at room temperature. The suspension was transferred to centrifuge tubes and centrifuged for 10 mins at 3000 rpm set at 4°C. The supernatant was diluted with methanol to achieve a volume of 10 mL and kept on ice (supernatant A). Each plant was extracted twice.

*Quantitative determination of free gallic acid*

200 μL of supernatant A was transferred to a test tube (4 tube/sample) and dried under vacuum pressure. 600 μL of sulphuric acid (0.2 N) was then added to the tubes. To three tubes, 900 μL of rhodanine solution (0.667%) and to the fourth tube 900 μL of methanol was added. The fourth tube was set as a blank. After 9 min 600 µL of potassium hydroxide solution (0.5 N) was added to all of the tubes. 6 mins later 12.9 mL of distilled water was also added. After 25 min the absorbance of the red-purple solution was measured at 520 nm against the blank (18). 

In order to quantify the amount of free gallic acid, its calibration curve was determined. Five different concentrations (4-20 µg/5 mL) of gallic acid were used to prepare the calibration curve. 

*Data analysis*

Data analysis was performed using SPSS, Tukey-post test and p < 0.05 was considered as being significant.

## Results and Discussion

In this research, the UV-spectroscopy method using rhodanin as the reagent was used in order to assay the free gallic acid. The principle of this method was the formation of a complex between rhodanine and gallic acid with a maximum absorbance at 520 nm. Rhodanine reacts with the vicinal hydroxyl groups of gallic acid and forms a reddish complex. The unreacted rhodanine in basic solution has no absorbance at wavelengths of higher than 450 nm. In this way the gallic acid-rhodanine complex can be conveniently determined with no interference. Rhodanine also reacts with quinines and hydroquinones, but the reaction products show no absorption at longer wavelengths (19). This method is reliable for quantitative routine analysis, very specific and has no interference with other plant phenolic compounds including tannic acid. As such it presents high sensitivity and precision (18).

The calibration curve of gallic acid was linear over the tested concentrations (4-20 µg/5mL) (Y = 0.0271X + 0.0232, r^2 ^= 0.9935).

The results of the gallic acid content in Triphala’s fruits are illustrated in Table 1.

**Table 1:** Gallic acid content in Triphala's fruits

**Plant name**	**Sample**	Gallic acid percentage*
*Phyllantus embelica*	1	1.96 ± 0.09
	2	1.79 ± 0.07
	3	2.18 ± 0.17
*Terminalia chebula* (Black)	1	0.28 ± 0.01
	2	0.80 ± 0.08
	3	0.43 ± 0.02
*Terminalia chebula* (Yellow)	1	0.56 ± 0.01
	2	0.37 ± 0.03
	3	0.32 ± 0.02
*Terminalia bellerica*	1	0.79 ± 0.05
	2	0.93 ± 0.04
	3	1.01 ± 0.04

* data are mean ± SD of six sample

The results showed that the gallic acid content differs in Triphala’s fruits. *Phyllanhus emblica* contained the highest amount of gallic acid (1.79-2.18%) followed by *Terminalia bellerica* (0.79-1.01%) and *Terminalia chebula* (0.28-0.80%) contained the least. In addition, the percentage of gallic acid in samples purchased from different markets showed significant differences (p < 0.05). The difference between the samples of *Phyllanhus emblica* is comparatively low (about 20%) but the difference in the gallic acid content among the three collected samples of black-*Terminalia chebula* was relatively high (280%). Gallic acid has a tannin structure that is very sensitive to oxidation; as such unsuitable storage conditions (with regards to temperature and humidity) and long storage periods can lead to its destruction (17). Therefore, the difference between the samples of each plant may be due to their different origin or storage conditions. As the difference between the *T. chebula* samples was higher than the others, it could be concluded that the *T. chebula* components are more sensitive to storage conditions or that the difference between the plant components from different sources is highly considerable.

In a separate research study, the percentage of gallic acid in the fruits comprising Triphala in India was determined using the HPLC technique. This research showed that the gallic acid percentage in *T. bellerica* was higher than the other fruits and that *Phyllanhus emblica*contained the lowest amount of this compound (9). These results are different from those of the recent study. The difference is most likely due to the origin and storage condition of the plants as was discussed before. It could be concluded that as Triphala’s three fruits are of importance and do not grow in Iran, when producing standard formulations importance should be placed on certain factors such as origin, standard storage conditions and expiration date. Moreover, the rhodanine assay based on spectrophotometry is one of the best methods for the standardization of Triphala due to its simplicity, reproducibility, low cost and ease of application.
